# Presentation of Autoantigen in Peripheral Lymph Nodes Is Sufficient for Priming Autoreactive CD8^+^ T Cells

**DOI:** 10.3389/fimmu.2017.00113

**Published:** 2017-02-10

**Authors:** Nadine Honke, Namir Shaabani, John R. Teijaro, Urs Christen, Cornelia Hardt, Judith Bezgovsek, Philipp A. Lang, Karl S. Lang

**Affiliations:** ^1^Medical Faculty, Institute of Immunology, University of Duisburg-Essen, Essen, Germany; ^2^Clinic of Gastroenterology, Hepatology and Infectious Diseases, Heinrich-Heine-University, Düsseldorf, Germany; ^3^Department of Immunology and Microbial Science, The Scripps Research Institute, La Jolla, CA, USA; ^4^Pharmazentrum Frankfurt, Goethe University Hospital Frankfurt, Frankfurt am Main, Germany; ^5^Medical Faculty, Department of Molecular Medicine II, Heinrich-Heine-University Düsseldorf, Düsseldorf, Germany

**Keywords:** autoimmune diabetes, lymph nodes, LCMV, enforced viral replication, sphingosine-1-phosphate receptor

## Abstract

Peripheral tolerance is an important mechanism by which the immune system can guarantee a second line of defense against autoreactive T and B cells. One autoimmune disease that is related to a break of peripheral tolerance is diabetes mellitus type 1. Using the RIP-GP mouse model, we analyzed the role of the spleen and lymph nodes (LNs) in priming CD8^+^ T cells and breaking peripheral tolerance. We found that diabetes developed in splenectomized mice infected with the lymphocytic choriomeningitis virus (LCMV), a finding showing that the spleen was not necessary in generating autoimmunity. By contrast, the absence of LNs prevented the priming of LCMV-specific CD8^+^ T cells, and diabetes did not develop in these mice. Additionally, we found that dendritic cells are responsible for the distribution of virus in secondary lymphoid organs, when LCMV was administered intravenously. Preventing this distribution with the sphingosine-1-phosphate receptor antagonist FTY720 inhibits the transport of antigen to peripheral LNs and consequently prevented the onset of diabetes. However, in case of subcutaneous infection, administration of FTY720 could not inhibit the onset of diabetes because the viral antigen is already presented in the peripheral LNs. These findings demonstrate the importance of preventing the presence of antigen in LNs for maintaining tolerance.

## Introduction

The immune system is divided structurally into primary lymphoid organs (PLOs) and secondary lymphoid organs (SLOs). PLOs include the thymus and bone marrow, which are responsible for the generation and differentiation of B and T cells; SLOs include the spleen and lymph nodes (LNs) in addition to the organized lymphoid tissues associated with mucosal membranes, such as the tonsils and Peyer’s patches. SLOs provide an optimal setting for effective antigen presentation and initiation of immune responses (1), whereas PLOs guarantee central tolerance that leads to deletion of autoreactive T and B cells (2). The SLOs provide a second line of defense and guarantee peripheral tolerance that suppresses the autoreactive T cells that escape from central tolerance in the thymus (2). Any factors disturbing this mechanism can establish autoimmune diseases such as type 1 diabetes (3).

The results of several experimental studies have shown that the encounter between antigen and naive T cells does not occur outside of organized lymphoid tissues (4–6) probably because T cells do not migrate to non-lymphoid tissues without infection due to the absence of homing receptors (7). The structure of the spleen provides optimal conditions for inducing the innate and adaptive immune response. In particular, the spleen captures antigen and guarantees sufficient viral replication to activate the adaptive immune system (8, 9). Interestingly, absence of spleen has only a slight impact on the antiviral immune response which can be compensated by other lymphoid organs (10), although splenectomy in the childhood has a big hazard of overwhelming infection and high susceptibility to bacterial infection (11).

Although LNs share with spleen its function as lymphoid organs and the ability to prime CD8^+^ T cells (12), but they differ structurally, as they possess a specialized vascular and lymphatic systems including high endothelial venules (HEVs), which considered the main sites of lymphocyte entry from the blood (13). Deletion of LNs leads to a dramatic defect in immune system (14). Additionally, skin graft transplantation experiments showed that applying antigen can only activate the immune system when afferent lymphatic vessels and draining LNs were intact (15). Moreover, a previous study from Gagnerault et al. showed that pancreatic LNs are precisely required for priming of β-cell autoreactive T cells (16). These results raise the question whether presentation of autoantigen in peripheral LNs is sufficient to break peripheral tolerance and for the onset of autoimmune diseases.

In this study using the lymphocytic choriomeningitis virus (LCMV), we found that early viral distribution in peripheral LNs is very important for guaranteeing efficient priming of CD8^+^ T cells.

## Research Design and Methods

### Mice

All experiments were performed with animals housed in single ventilated cages, under the authorization of the Veterinäramt Nordrhein Westfalen (Düsseldorf, Germany) and in accordance with the German law for animal protection. All mice were sex matched, and they were used 8–10 weeks old. RIP-GP mice (17), which express the LCMV glycoprotein as a transgene under the rat insulin promoter, were used for the analysis of autoimmune diabetes. P14/CD45.1 mice expressing a T cell receptor specific for LCMV glycoproteins 33–41 (LCMVeGP33–41) as a transgene were used for adoptive transfer experiments (18). Aly/aly mice (19) and aly/aly × RIP-GP mice were used to study the role of LNs in priming CD8^+^ T cells. CD11c-diphtheria toxin receptor (DTR) mice (20) and CD169-DTR mice (21) were used to investigate the role of dendritic cells (DCs) (CD11c^+^) or metallophilic marginal zone macrophages (CD169^+^) in viral transport. All mice were maintained on a C57BL/6 background.

### Diphtheria Toxin Treatment

Dendritic cells of CD11c-DTR mice were depleted as described previously (22). Briefly, diphtheria toxin was injected intraperitoneally (i.p.) at a dose of 15 µg/kg body weight (bw) daily starting from day 0. CD169^+^ macrophages of CD169-DTR mice were injected intraperitoneally (i.p.) at a dose 30 µg/kg bw on days −3, 0, 1, and 2 because the recovery of CD169^+^ cells needs longer than cells in CD11c-DTR mice as it was previously described (21, 23).

### Bone Marrow Chimeras

To generate bone marrow chimeras, we irradiated aly/aly × RIP-GP and RIP-GP mice with 9.5 Gy (320 kV X-rays, 3 Gy/min, 0.35 mm copper + 1.5 mm aluminum filter; Pantak-Seifert, Ahrensburg, Germany). On the next day, we transferred 10^7^ bone marrow cells from wild-type (WT) mice into the irradiated mice. After 15 days, we administered clodronate liposomes (24) to ensure macrophage exchange in WT > RIP-GP and WT > aly/aly × RIP-GP chimeras. LCMV infection was performed after 30 days.

### Splenectomy

Mice were anesthetized with ketamine (4 mg/mouse; CEVA, Düsseldorf, Germany) and xylazine (0.23 mg/mouse; CEVA). A midline laparotomy incision was made, and the spleen was removed after ligation of the blood vessels. Sham mice were used as controls.

### Virus and Plaque Assay

Lymphocytic choriomeningitis virus WE, originally obtained from F. Lehmann-Grube (Heinrich Pette Institute, Hamburg, Germany) was propagated in L929 cells. Mice were infected i.v. with LCMV at the indicated doses. Viral titers were measured in a plaque-forming assay using MC57 cells, as previously described (25).

### FTY720 Treatment

Mice were treated daily by gavage at a dose of 1 mg/kg bw FTY720 (Sigma-Aldrich, Steinheim, Germany) starting on day −2 or 3.

### Flow Cytometry

Tetramers were provided by the National Institutes of Health (NIH, Bethesda, MD, USA) Tetramer Facility. Spleen, inguinal lymph nodes (iLNs), or blood were stained with allophycocyanin-labeled GP33 major histocompatibility complex class I tetramers (GP33/H-2Db) for 15 min at 37°C. After incubation, the samples were stained with anti-CD8 (eBiosciences, Franklin Lakes, NJ, USA) for 30 min at 4°C. Erythrocytes were then lysed with 1 ml BD lysing solution (BD Biosciences, Franklin Lakes, NJ, USA). Absolute numbers of GP33-specific CD8^+^ T cells per microliter of blood were calculated by fluorescence-activated cell sorting (FACS) using fluorescing beads (BD Biosciences). For intracellular staining of LCMV-NP in DCs, spleen was digested with DNase I (0.2 mg/ml, Sigma-Aldrich, Steinheim, Germany) and Liberase Collagenase 1 and 2 (0.05 mg/ml, Roche, Penzberg, Germany). Cells were permealized with Saponin (0.01%, Sigma-Aldrich) and stained with house-made LCMV-NP (VL4) antibody.

### Total RNA Extraction, cDNA Synthesis, and Quantitative Real-time Polymerase Chain Reaction

RNA was isolated from liver, spleen, kidney, lung, and iLNs with the RNA Mini Kit (Qiagen, Hilden, Germany). Quantitation of RNA was performed with a NanoDrop ND-1000 spectrophotometer (Thermo Scientific, Wilmington, DE, USA). The RNA was reverse transcribed to cDNA with the Quantitect Reverse Transcription kit (Qiagen). Gene expression analysis was performed with assays from Eurofins [LCMV nucleoprotein (NP), and LCMV glycoprotein (GP)].

### Blood Glucose Measurement

Serum glucose concentrations were measured with a contour meter (Bayer, Leverkusen, Germany). Mice were considered diabetic if the glucose concentration was higher than 200 mg/dl.

### Lymphocyte Transfer

We labeled 10^7^ splenocytes from P14 mice expressing CD45.1 with 1 µM carboxyfluorescein succinimidyl ester (CFSE; Invitrogen, Carlsbad, CA, USA) and injected them intravenously into aly/aly or C57BL/6 control mice. The proliferation of P14 T cells was assessed in blood and spleen by CFSE dilution and flow cytometry.

### Histology

Immunofluorescence and conventional histology studies were performed as previously described (26). For immunofluorescence histology of the pancreas, snap-frozen tissue was stained with primary antibody anti-insulin (Dako, Hamburg, Germany) and then with secondary antibody anti-guinea pig (Jackson ImmunoResearch, West Grove, PA, USA) for the detection of insulin-producing β-islet cells and with anti-CD8 (eBioscience) for staining of cytotoxic CD8^+^ T cells. For the immunofluorescence histology of the spleen, snap-frozen tissue was either stained with LCMV nucleoprotein (VL-4, green, made in-house) and/or for metallophilic marginal zone macrophages (CD169^+^, red, eBioscience)/DCs (CD11c^+^, red, eBioscience) and B cells (B220, blue, eBioscience)/T cells (CD90.2, blue, eBioscience). For conventional immunohistochemistry, snap-frozen tissue was stained with rat anti-mouse polyclonal antibody to LCMV nucleoprotein (VL4, made in-house). Polyclonal anti-rat biotin antibody (eBioscience) and anti-biotin streptavidin–peroxidase (Thermo Fisher Scientific) were then used before visualization with a 2-solution 3,3′-diaminobenzidine (DAB) staining kit (Invitrogen).

### Statistical Analysis

If not otherwise stated, data are expressed as means plus or minus SEM. Student’s *t*-test was used to detect statistically significant differences between groups, and the log-rank (Mantel–Cox) test was used to detect statistically significant differences in the incidence of diabetes. Significant differences between several groups were detected by two-way analysis of variance. Statistical significance was set at the level of *P* < 0.05.

## Results

### Route of LCMV Infection Influences the Viral Titer Kinetic and CD8^+^ T Cell Number in Blood

**Figure 1:**
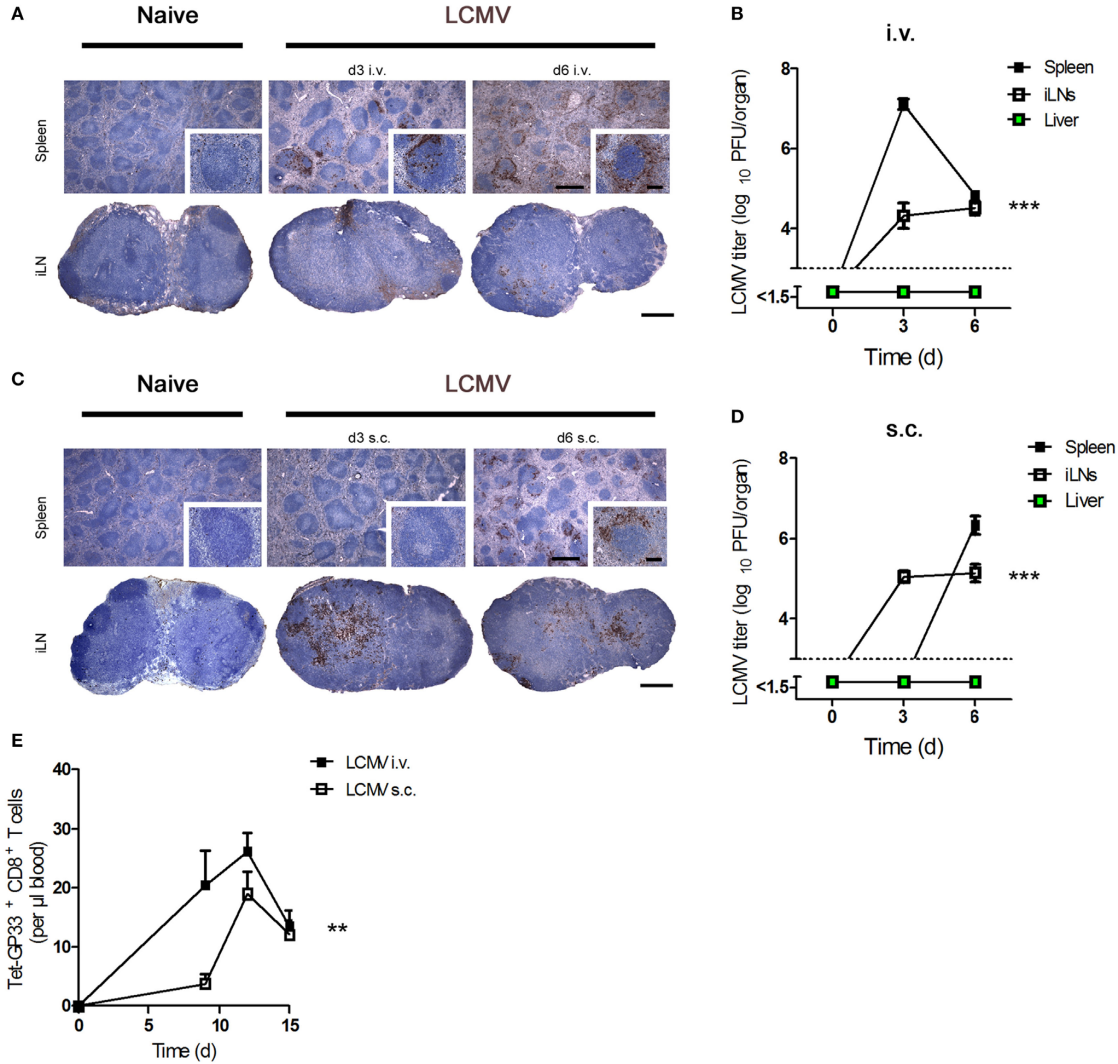
**Route of lymphocytic choriomeningitis virus (LCMV) infection influences the viral titer kinetic and CD8^+^ T number in blood**. **(A,B)** C57BL/6 mice were infected intravenously (i.v.) with 200 plaque-forming units (PFUs) lymphocytic choriomeningitis virus strain WE (LCMV-WE). **(A)** Conventional immunohistochemistry of LCMV in the spleen and inguinal lymph nodes (iLNs) on day 3 (d3) and day 6 (d6) after infection (*n* = 3). Scale bars on main images represent 500 µm, and scale bars on inlays represent 100 µm. Histological images were captured at 4× magnification (main images) or 20× magnification (inlays) with a Keyence BZ-9000E microscope. One of the three representative images is shown. **(B)** Viral titers in the spleen, iLNs, and liver were measured at the indicated time points (*n* = 7, pooled from two experiments). Black squares = spleen; white squares = iLNs; green squares = liver. **(C,D)** C57BL/6 mice were infected subcutaneously (s.c.) with 200 PFU LCMV-WE. **(C)** Conventional histological staining of LCMV in the spleen and iLNs at the indicated time points (d3 and d6). Scale bars on main images represent 500 µm, and scale bars on inlays represent 100 µm (*n* = 3). Histological images were captured at 4× magnification (main images) or 20× magnification (inlays) with a Keyence BZ-9000E microscope. One of the three representative images is shown. **(D)** Viral titers in the spleen, iLNs, and liver were measured on d3 and d6 after subcutaneously infection (*n* = 7; pooled from two experiments). Black squares = spleen; white squares = iLNs; green squares = liver. **(E)** C57BL/6 mice were intravenously (i.v.) or subcutaneously (s.c.) infected with 200 PFU LCMV-WE. Tetramer (Tet) GP33^+^ CD8^+^ T cells in the blood were counted on d9, d12, and d15 after infection (*n* = 7; pooled from two experiments). Black squares = LCMV-WE i.v.; white squares = LCMV-WE s.c. Statistical significance was set at the level of *P* < 0.05 and was determined by two-way analysis of variance **(B,D,E)**. **P < 0.01, ***P < 0.001.

First, we determined viral replication in the spleen and peripheral iLNs after administration of virus by various routes of infection. Intravenous (i.v.) infection of C57BL/6 mice with LCMV strain WE [lymphocytic choriomeningitis virus strain WE (LCMV-WE); 200 plaque-forming units (PFUs)] led to high levels of viral replication in the spleen and LNs 3 days after infection (Figures 1A,B). As expected, a dose of LCMV ≤200 PFU does not lead to viral replication in the liver (9, 27). In contrast, subcutaneous (s.c.) infection of C57BL/6 mice with the same dose of LCMV caused higher viral replication in LNs at day 3 but delayed viral titers in the spleen (Figures 1C,D). In line with these results, we found that LCMV-specific CD8^+^ T cell number in blood was higher after i.v. infection compared to s.c. infection (Figure 1E). We conclude that viral titer kinetics and CD8^+^ T cell number in the blood are influenced by the route of infection.

### DCs Are the Cardinal Contributors to the Transport of Antigen to LNs

**Figure 2:**
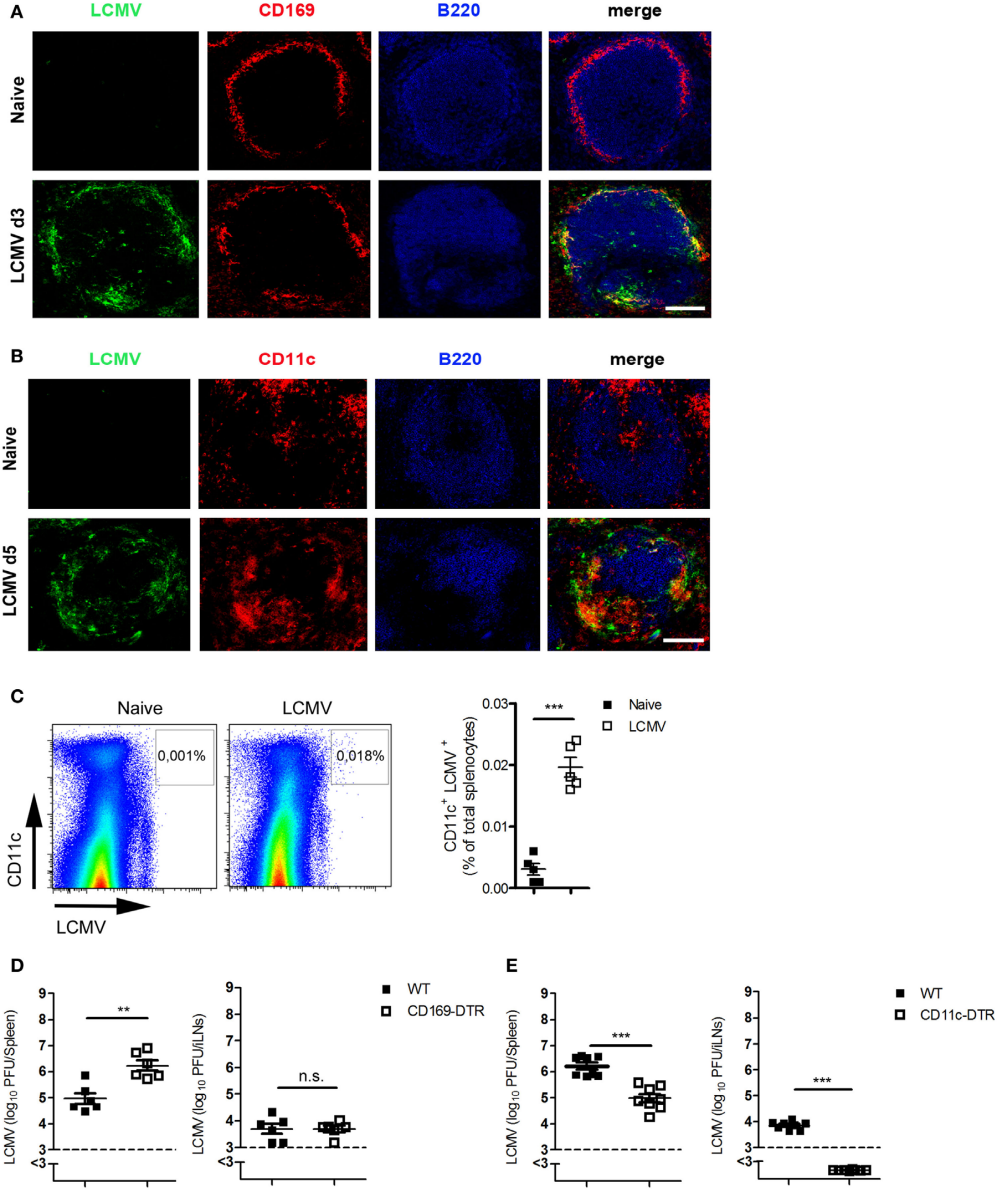
**Dendritic cells (DCs) are the cardinal contributors of the transport of antigen to lymph nodes**. **(A,B)** C57BL/6 mice were infected intravenously (i.v.) with 200 plaque-forming units (PFUs) of lymphocytic choriomeningitis virus strain WE (LCMV-WE). Immunofluorescent sections of spleen were stained for **(A)** LCMV nucleoprotein (LCMV-NP; green), CD169^+^ macrophages (CD169; red), and B cells (B220; blue) on day 3 after infection or for **(B)** LCMV-NP (green), DCs (CD11c; red), and B cells (B220; blue) on day 5 (d5) after infection. Scale bars represent 100 µm (*n* = 3). Fluorescence images were captured at 20× magnification with a Keyence BZ-9000E microscope. One of the three representative images is shown. **(C)** C57BL/6 mice were infected i.v. with 2 × 10^6^ PFU LCMV-WE. Virus-positive DCs were measured in spleen tissue on day 3 after infection by fluorescence-activated cell sorting (*n* = 5, from one experiment). Black squares = naive; white squares = LCMV. **(D)** CD169-diphtheria toxin receptor (DTR) and littermate control mice were treated with diphtheria toxin [30 µg/kg body weight (bw)] on days −3, 0, 1, and 2. On day 0, mice were infected with 200 PFU LCMV-WE. Viral titers were measured in the spleen and iLNs on d3 after infection (*n* = 6, pooled from two experiments). Black squares = wild type (WT); white squares = CD169-DTR. **(E)** CD11c-DTR and littermate control mice (WT) were treated daily with diphtheria toxin (15 µg/kg bw) from day 0 until day 2. On day 0, mice were infected with 2 × 10^6^ PFU LCMV-WE. Viral titers were measured in the spleen and inguinal lymph nodes (iLNs) on d3 after infection (*n* = 8, pooled from two experiments). Black squares = WT; white squares = CD11c-DTR. Statistical significance was set at the level of *P* < 0.05 and was determined by Student’s *t*-test **(C–E)**. n.s., not significant; **P < 0.01, ***P < 0.001.

Next, we investigated which cells are responsible for the transfer of viral antigen to or between LNs. Immunofluorescent staining of spleen sections showed that most of the virus was co-localized with CD169^+^ macrophages (Figure 2A) and with CD11c^+^ cells (Figure 2B). FACS analysis showed also that most of the LCMV was found in CD11c^+^ cells (Figure 2C). To determine whether CD169^+^ macrophages, CD11c^+^ DCs, or both are responsible for viral transfer, we used CD169-DTR mice or CD11c-DTR mice. Treating these mice with diphtheria toxin depletes CD169^+^ cells or DCs, respectively (Figures S1 and S2 in Supplementary Material). CD169^+^ cells exerted no influence on antigen transfer because viral titer was higher in CD169-DTR mice than in WT mice in the spleen and not significantly different in iLNs (Figure 2D). By contrast, CD11c-DTR mice exhibited lower viral titers in the spleen in comparison to WT mice, whereas no viral antigen could be detected in LNs when DCs were absent (Figure 2E). From these data, we conclude that DCs contribute to the transfer of virus to LNs.

### The Spleen Is Not Necessary in Generating Autoimmunity

**Figure 3:**
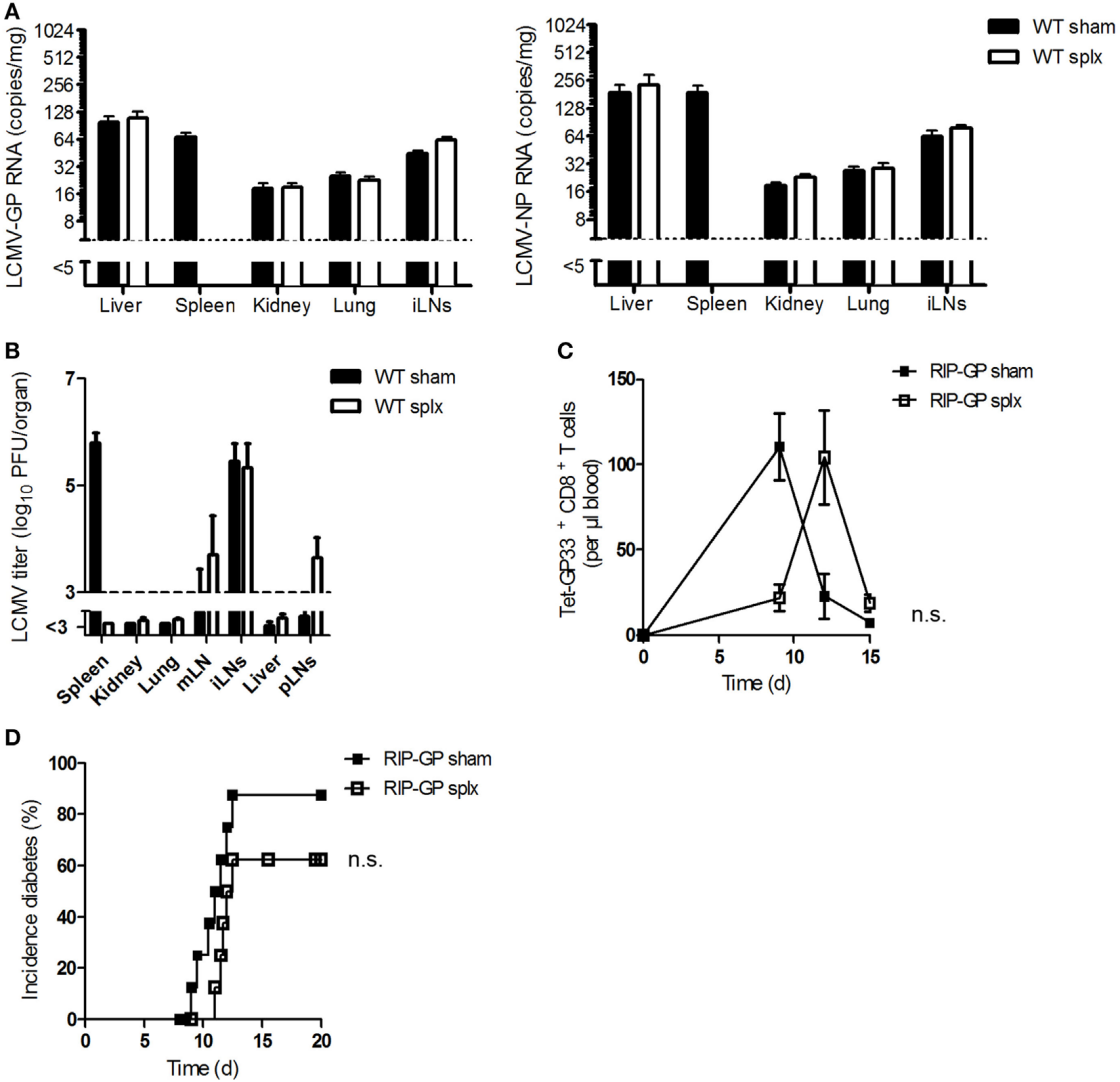
**The spleen is not necessary in generating autoimmunity**. **(A)** Sham and splenectomized (splx) C57BL/6 wild-type (WT) mice were infected intravenously with 2 × 10^6^ plaque-forming units (PFUs) of lymphocytic choriomeningitis virus strain WE (LCMV-WE). After 1 h, lymphocytic choriomeningitis virus (LCMV) glycoprotein (GP) or nucleoprotein (NP) copies were counted in the liver, spleen, kidney, lung, and iLNs by quantitative real-time polymerase chain reaction (qRT-PCR) (*n* = 4, from one experiment). Black column = WT sham; white column = WT splx. **(B)** Sham and splenectomized C57BL/6 mice were intravenously infected with 20 PFU of LCMV-WE. After 6 days, viral titers were measured in the indicated organs (*n* = 8; pooled from two experiments). Black column = WT sham; white column = WT splx. mLN, mesenteric lymph node; iLNs, inguinal lymph nodes and pLNs, pancreatic lymph nodes. **(C,D)** Transgenic splenectomized and sham mice expressing the LCMV-GP under control of the rat insulin promoter (RIP-GP mice) were infected with 200 PFU LCMV-WE. **(C)** Tetramer (Tet) GP33^+^ CD8^+^ T cells in the blood were counted at the indicated time points (*n* = 8–9, pooled from two experiments). Black squares = RIP-GP sham; white squares = RIP-GP splx. **(D)** The onset of diabetes was monitored (*n* = 9–10, pooled from two experiments). Black squares = RIP-GP sham; white squares = RIP-GP splx. Statistical significance was set at the level of *P* < 0.05 and was determined by two-way analysis of variance **(C)** and log-rank (Mantel–Cox) test **(D)**. n.s., not significant.

Previous results raised the question whether LNs alone, without assistance from the spleen, could prime CD8^+^ T cells. To answer this question, we first investigated the impact of the spleen on viral uptake by LNs or on viral replication in LNs. Viral uptake in WT splenectomized (splx) and control mice (sham) was measured. Viral copy numbers (Figure 3A) and viral titers (Figure 3B) were similar in both groups, a finding indicating that viral replication in LNs does not depend on the spleen.

To investigate the activation of autoreactive CD8^+^ T cells and the onset of diabetes, we infected transgenic splenectomized and sham mice expressing the LCMV glycoprotein under control of the rat insulin promoter (RIP-GP) (as controls) with LCMV. Surprisingly, after infection splenectomized mice exhibited strong but delayed priming of autoreactive CD8^+^ T cells in comparison to control mice (Figure 3C). This finding indicates that removing the spleen does not prevent the onset of diabetes (Figure 3D). The delay in CD8^+^ T cell activation in splenectomized mice may be due to the reduction of autoreactive T cells in these mice. Therefore, we conclude that the spleen is not necessary in generating autoimmunity.

### Presence of LNs and Organized Splenic Structure Are Essential for the Onset of Diabetes

**Figure 4:**
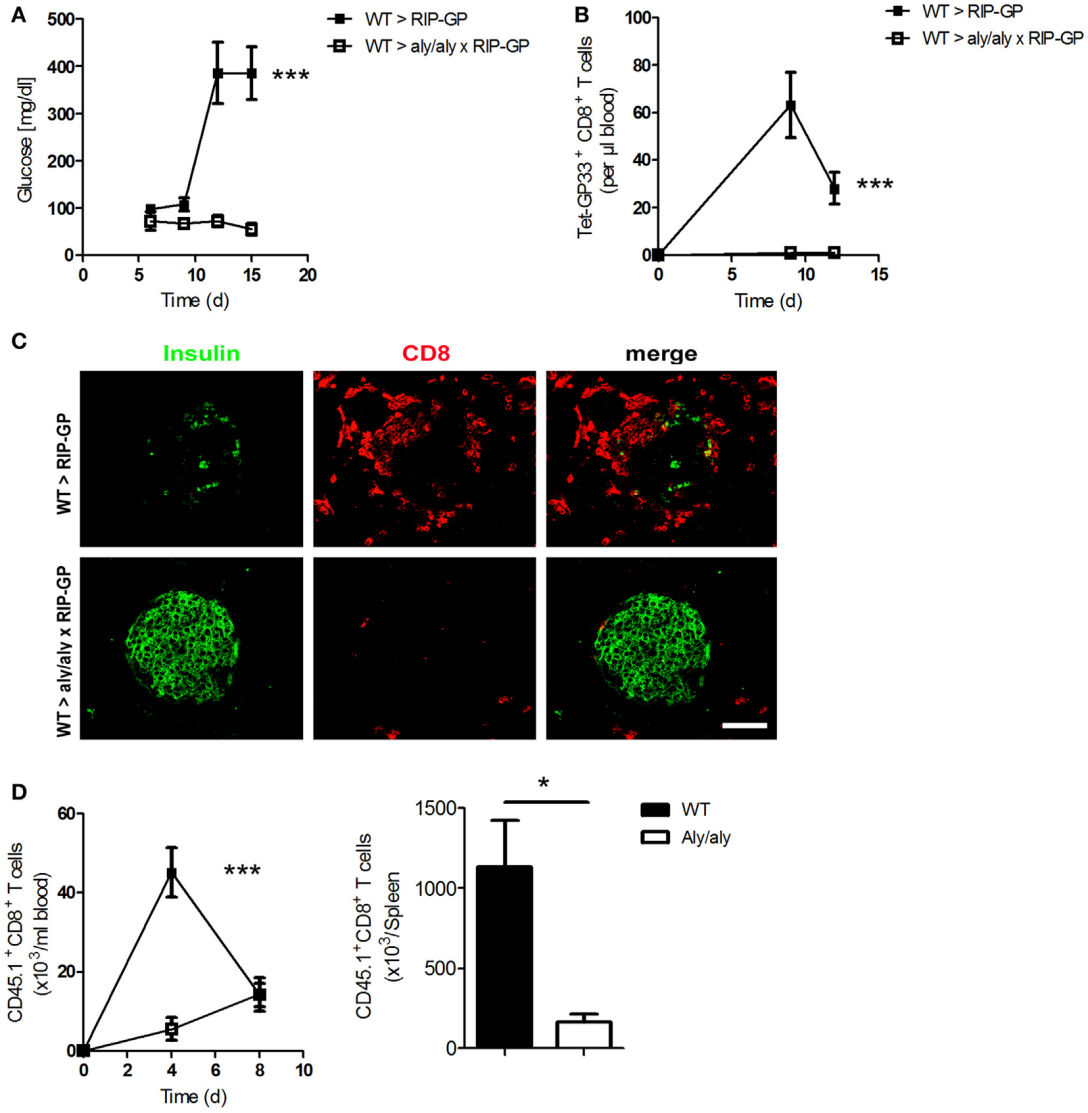
**Presence of lymph nodes (LNs) and organized splenic structure are essential for onset of diabetes**. **(A–C)** Transgenic mice expressing the lymphocytic choriomeningitis virus (LCMV) glycoprotein (GP) under control of the rat insulin promoter (RIP-GP mice) and homozygous double transgenic mice lacking LNs [alymphoplasia/alymphoplasia mice (aly/aly)] and crossed with RIP-GP mice (aly/aly × RIP-GP mice) were lethally irradiated. One day later, their immune systems were reconstituted with 10^7^ bone marrow cells from C57BL/6 wild-type (WT) mice. Thirty days later, mice were infected with 200 plaque-forming units (PFUs) of LCMV. **(A)** Serum glucose concentrations were determined on day 6 (d6), d9, d12, and d15 after infection (*n* = 4, from one experiment). Black squares = WT > RIP-GP; white squares = WT > aly/aly × RIP-GP. **(B)** Tetramer (Tet) GP33-specific CD8^+^ T cells in the blood were counted by flow cytometry at the indicated time points after LCMV infection (*n* = 4, from one experiment). Black squares = WT > RIP-GP; white squares = WT > aly/aly × RIP-GP. **(C)** Immunofluorescence histology of pancreas sections stained for insulin (green) and CD8^+^ T cells (red) on d6 after infection (*n* = 3; scale bar, 50 µm). Fluorescence images were captured at 20× magnification with a Keyence BZ-9000E microscope. One of the three representative images is shown. **(D)** 10^7^ splenocytes transferred from P14 × CD45.1 mice into WT and aly/aly mice. After 24 h, mice were infected with 200 PFU LCMV, and CD45.1^+^/CD8^+^ T cells were counted in blood on day 4 (d4) and day 8 (d8) and in spleen day 8 (d8) after infection (*n* = 3, from one experiment). Black squares or column = WT; white squares or column = aly/aly. Statistical significance was set at the level of *P* < 0.05 and was determined by two-way analysis of variance [**(A,B,D)** (left)] or Student’s *t*-test [**(D)** (right)]. *P < 0.5; ***P < 0.001.

To study the role of LNs in the incidence of diabetes, we crossed mice lacking LNs [alymphoplasia/alymphoplasia (aly/aly)] with RIP-GP mice to get homozygous double mutants mice. Aly/aly mice have additional defects in other immune cells, such as B and T cells (10, 28). To overcome some of these defects, we generated bone marrow chimeras and transferred bone marrow from WT mice into aly/aly × RIP-GP mice. After reconstitution, we infected these mice intravenously (i.v.) with the diabetes-inducing dose of LCMV-WE and monitored the onset of diabetes. Surprisingly, diabetes did not develop in these mice, whereas control mice exhibited elevated glucose levels (Figure 4A). This phenotype was associated with the absence of virus-specific CD8^+^ T cells in the mice lacking LNs (Figure 4B). Moreover, the infiltration of CD8^+^ T cells into the pancreas was blunted, and β-islet cells were not destroyed because the LNs were absent (Figure 4C).

To investigate the defect in the priming of CD8^+^ T cells in the absence of LNs, we transferred splenocytes from P14/CD45.1 mice into WT and aly/aly mice and infected both groups, with LCMV. We measured the number of transferred CD45.1^+^ CD8^+^ T cells and found that these T cells proliferate much more stronger in WT mice than in aly/aly mice (Figure 4D). Taking together, these results with the fact that aly/aly mice have architectural defect in the spleen, even with the bone marrow chimera strategy as shown by Karrer et al. (10), we conclude that peripheral LNs and organized structure of the spleen play an essential role in priming of peripheral CD8^+^ T cells and affecting the maintenance of tolerance.

### FTY720 Prevents Viral Transfer to LNs and Thereby Inhibits the Onset of Diabetes

**Figure 5:**
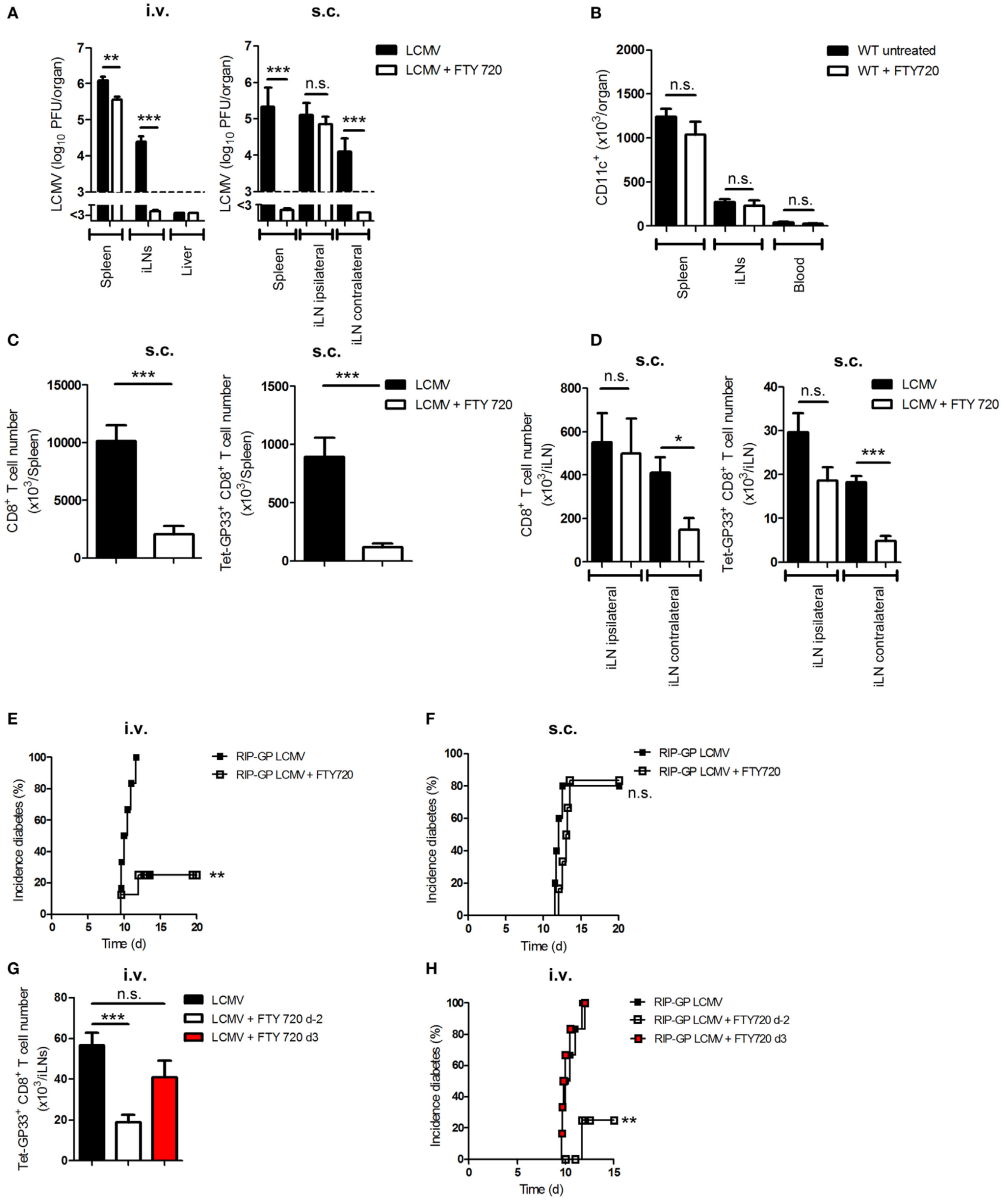

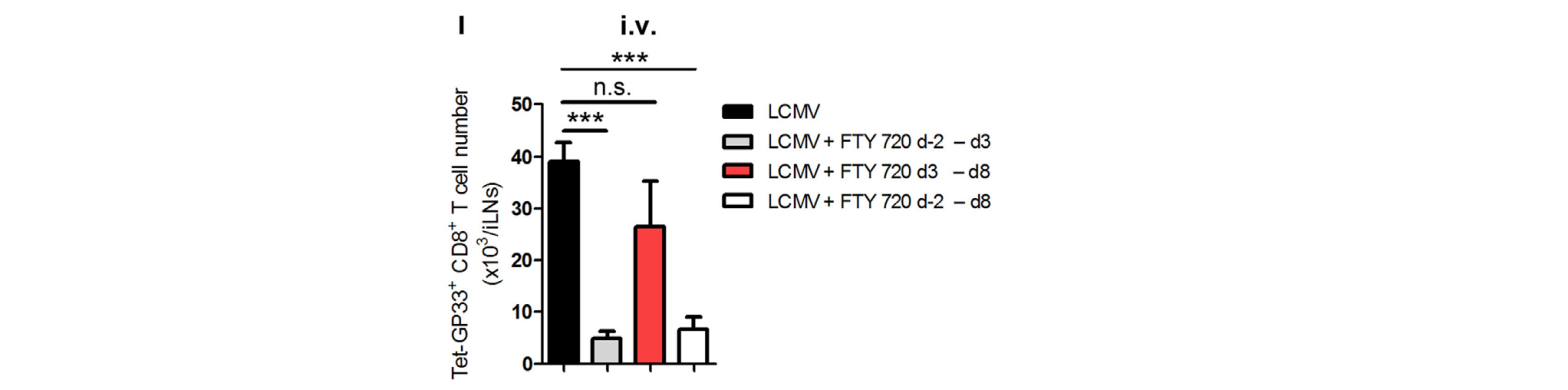
**FTY720 prevents viral transfer to lymph nodes and thereby inhibits the onset of diabetes**. **(A)** C57BL/6 wild-type (WT) mice were treated daily with a sphingosine-1-phosphate antagonist [FTY720; 1 mg/kg body weight (bw)] starting from day −2 or were left untreated. Mice were infected intravenously (i.v.) or subcutaneously (s.c.) with 20 plaque-forming units (PFUs) of lymphocytic choriomeningitis virus strain WE (LCMV-WE). Viral titer was measured in the spleen, inguinal lymph nodes (iLNs) and liver on day 3 (d3; i.v.) or day 6 (d6; s.c.) after infection (*n* = 6; pooled from two experiments). Black column = LCMV; white column = LCMV + FTY720. **(B)** C57BL/6 WT mice were treated daily with FTY720 (1 mg/kg bw) starting from day −2 or were left untreated. CD11c^+^ cells in the spleen, iLNs, and blood were counted on day 0 (*n* = 6, pooled from two experiments). Black column = WT untreated; white column = WT + FTY720. **(C,D)** C57BL/6 WT mice were treated daily with FTY720 (1 mg/kg bw) starting on day −2 or were left untreated. Mice were infected s.c. with 20 PFU LCMV-WE. On day 12, total and LCMV-specific CD8^+^ T cells were counted in the spleen **(C)** and iLNs **(D)** (*n* = 6, pooled from two experiments). Black column = LCMV; white column = LCMV + FTY720. **(E,F)** Transgenic mice expressing the lymphocytic choriomeningitis virus (LCMV) glycoprotein (GP) under control of the rat insulin promoter (RIP-GP) were treated daily with FTY720 (1 mg/kg bw) starting from day −2 or were left untreated. Mice were intravenously or subcutaneously infected with 20 PFU LCMV-WE. Onset of diabetes was measured (**E)** i.v., *n* = 6–8, pooled from two experiments; **(F)** s.c., *n* = 5–6, pooled from two experiments. Black squares = RIP-GP LCMV; white squares = RIP-GP LCMV + FTY720. **(G)** C57BL/6 WT mice were treated daily with a sphingosine-1-phosphate antagonist [FTY720; 1 mg/kg body weight (bw)] starting from day −2 or 3 of infection or left untreated. Mice were infected intravenously (i.v.) with 20 PFU of LCMV-WE. On day 12, tetramer (Tet) GP33-specific CD8^+^ T cells in iLNs were counted by flow cytometry (*n* = 8, pooled from two experiments). Black column = LCMV; white column = LCMV + FTY720 (starting day −2); red column = LCMV + FTY720 (starting d3). **(H)** Transgenic mice expressing the LCMV glycoprotein (GP) under control of the rat insulin promoter (RIP-GP) were treated daily with FTY720 (1 mg/kg bw) starting from day −2 or 3 of infection or were left untreated. Mice were intravenously infected with 20 PFU LCMV-WE. Onset of diabetes was measured (*n* = 6, pooled from two experiments). Black squares = RIP-GP LCMV; white squares = RIP-GP LCMV + FTY720 day −2; red squares = RIP-GP LCMV + FTY d3. **(I)** C57BL/6 WT mice were treated daily with a sphingosine-1-phosphate antagonist [FTY720; 1 mg/kg body weight (bw)] starting from day −2 till day 3, from day 3 till day 8, or day 2 till day 8 of infection or left untreated. Mice were infected intravenously (i.v.) with 20 PFU of LCMV-WE. On day 9, tetramer (Tet) GP33-specific CD8^+^ T cells in iLNs were counted by flow cytometry (*n* = 6, pooled from two experiments). Black column = LCMV; gray column = LCMV + FTY720 (starting day −2 till day 3); red column = LCMV + FTY720 (starting day 3 till day 8); white column = LCMV + FTY720 (starting day −2 till day 8). Statistical significance was set at the level of *P* < 0.05 and was determined by Student’s *t*-test **(A–D,G,I)** or log-rank (Mantel–Cox) test **(E,F,H)**. n.s., not significant; *P < 0.5; **P < 0.01; ***P < 0.001.

A previous study showed that the sphingosine-1-phosphate receptor antagonist (FTY720) can modulate the trafficking of DCs *in vivo* (29). To examine its influence on viral transfer, we infected C57BL/6 WT mice i.v. or s.c. with LCMV. Additionally, we treated one group of mice daily with FTY720 beginning on day −2 (untreated mice served as controls). Interestingly, FTY720 treatment inhibited the transfer of virus from spleen to iLNs in i.v.-infected mice or from ipsilateral iLN to spleen or contralateral iLNs in s.c.-infected mice (Figure 5A). The inhibition of antigen transfer was not due to a reduction in the number of DCs because FTY720 treatment has no effect on DC number without infection (Figure 5B). The prevention of viral transfer from LNs to the spleen after s.c. infection was associated with the absence of priming of total and virus-specific CD8^+^ T cells in the spleen and in the contralateral iLN but not in the ipsilateral iLN (Figures 5C,D).

We and other groups have shown that FTY720 treatment can prevent the onset of diabetes in RIP-GP mice infected i.v. with LCMV (Figure 5E) (30). We speculated that viral replication in LNs of RIP-GP mice after s.c. infection is sufficient to prime CD8^+^ T cells even during FTY720 treatment. Indeed, we found that the administration of FTY720 did not inhibit the onset of diabetes in s.c.-infected mice (Figure 5F). To prove that the prevention of onset of diabetes in i.v.-infected mice was due to the inhibition of antigen spreading to lymphoid organs and not due to a direct effect on T-lymphocytes circulation, we infected three groups of C57BL/6 mice intravenously with LCMV. Two groups were treated additionally with FTY720 starting from day −2 or from day 3 after infection. Administration of FTY720 before infection prevented viral spread and consequently led to less LCMV-specific CD8^+^ T cell number in the iLNs in comparison to the untreated group, whereas the group that received FTY720 starting from day 3 after infection and have already viral distribution in iLNs showed similar number of LCMV-specific CD8^+^ T cells to the untreated one (Figure 5G). To check whether late treatment of FTY720 is enough to onset autoimmune response, we treated RIP-GP mice with FTY720 starting either from day −2 or from day 3 after infection. We found that treating mice with FTY720 at later time point did not prevent the priming of autoreactive CD8^+^ T cells and the onset of diabetes (Figure 5H). Additionally, treating mice with FTY720 only between days −2 and 3 was enough to reduce virus-specific CD8^+^ T cell priming in the inguinal LNs (Figure 5I), which suggest that the effect of FTY720 is not direct on CD8^+^ T cells but on DCs. From these findings, we conclude that viral transfer is dependent on sphingosine-1-phosphate and that antigen replication in LNs is essential for priming peripheral CD8^+^ T cells and breaking immunological tolerance.

## Discussion

The results of this study suggest that presentation of autoantigen in peripheral LNs is sufficient for priming autoreactive CD8^+^ T cells and generating autoimmune disease. Here, we could show that the dendritic cell-dependent transport of autoantigen in LNs plays an important role in breaking peripheral tolerance. Administration of FTY720 prevents antigen spreading between lymphoid organs, which is essential for priming of autoreactive CD8^+^ T cells in peripheral LNs. It is still to be addressed whether the phenotype observed by Pinschewer et al. (30) showing a reduction of memory CD8^+^ T cells recruitment after FTY720 treatment was also due to the inhibition of antigen distribution or to lymphocyte arrest in lymphoid organs.

Although our study confirmed the finding which made by Karrer et al. showing the importance of LNs in activation the adaptive immune system (14), the current study does not exclude either the important role of the spleen in the immune system or the fact that most children born without a spleen die of infection during the first months after birth (31, 32). Moreover, the architectural defect in the spleen of aly/aly mice, even with the bone marrow chimera strategy as shown by Karrer et al. (10), make it difficult to assert that spleen alone is not enough to prime autoreactive T cells.

Oral administration of FTY 720 induces immune tolerance in mice through induction of regulatory T cells, inhibition of effector T cell responses, and DC trafficking (29). In this study, we found that the effect of FTY720 as immune tolerance inducer is depending on the route of infection. After i.v. infection with LCMV, FTY720 prevents the transfer of autoantigen to peripheral LNs and consequently inhibits the priming of autoreactive CD8^+^ T cells, whereas after s.c. infection, FTY720 exerts no prevention on onset of diabetes. Additional investigations are necessary for determining whether APC trafficking is completely inhibited or whether only the migration of autoantigen-carrying cells is influenced.

Interestingly, the onset of diabetes in non-obese diabetic mice was inhibited by continuous treatment with FTY720 (33). Withdrawal of treatment led to more rapid development of disease independent of the pancreatic lymph nodes. Our results may explain this phenotype, in which the prevention of autoantigen transport in LNs by the administration of FTY720 can inhibit the development of diabetes.

It is still to be investigated why LNs provide a better environment than the spleen. One explanation could be the location of LNs where they afford a better distribution of T cells. Another explanation may be due to viral load in the lymphoid organs, where we see that LNs have higher antigen amount than spleen if it is compared with size of the organ and consequently leads to better antigen–T cell affinity. The reason behind that may be the absence of red pulp macrophages in LNs but not in spleen which are strong IFN-I-dependent antiviral APCs (8). Or third, the presence of HEVs in LNs, but not in spleen (34), can provide an optimal lymphocyte trafficking.

Finally, our findings give an explanation why subcutaneously vaccination may be more efficient than intravenously antigen administration. The subcutaneous route of administration provide sufficient priming of T cells and simultaneously help to avoid undesirable systemic adverse effects, such as toxicity or vaccine-wide distribution in various organs such as the liver, where no interaction occurs between antigen and the adaptive immune system.

## Author Contributions

NH and NS designed the study, performed the experiments, analyzed the data, and wrote the manuscript. CH, helped in manuscript writing. PAL, JRT, JB, and UC discussed the results. KSL performed experiment of LCMV percentage in DCs and discussed the results.

## Conflict of Interest Statement

The authors declare that the research was conducted in the absence of any commercial or financial relationships that could be construed as a potential conflict of interest.
