# Supplementary figures and images for: Presentation of Autoantigen in Peripheral Lymph Nodes Is Sufficient for Priming Autoreactive CD8+ T Cells
Source: Front Immunol. 2017 Feb 10;8:113. doi: 10.3389/fimmu.2017.00113 (PMC5301005; doi:10.3389/fimmu.2017.00113)

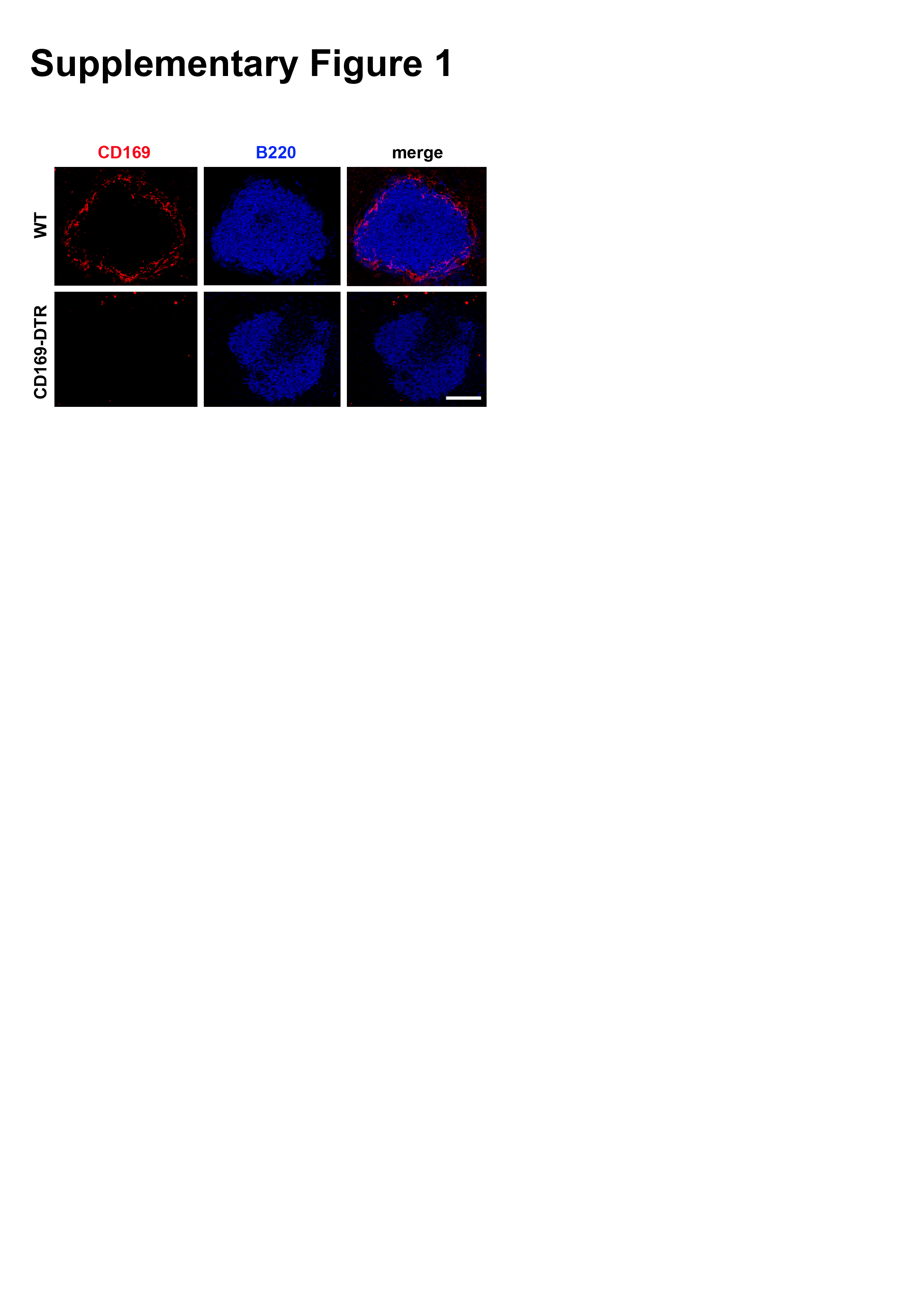

Supplement: Supplementary file 1 [file image_1.jpeg]

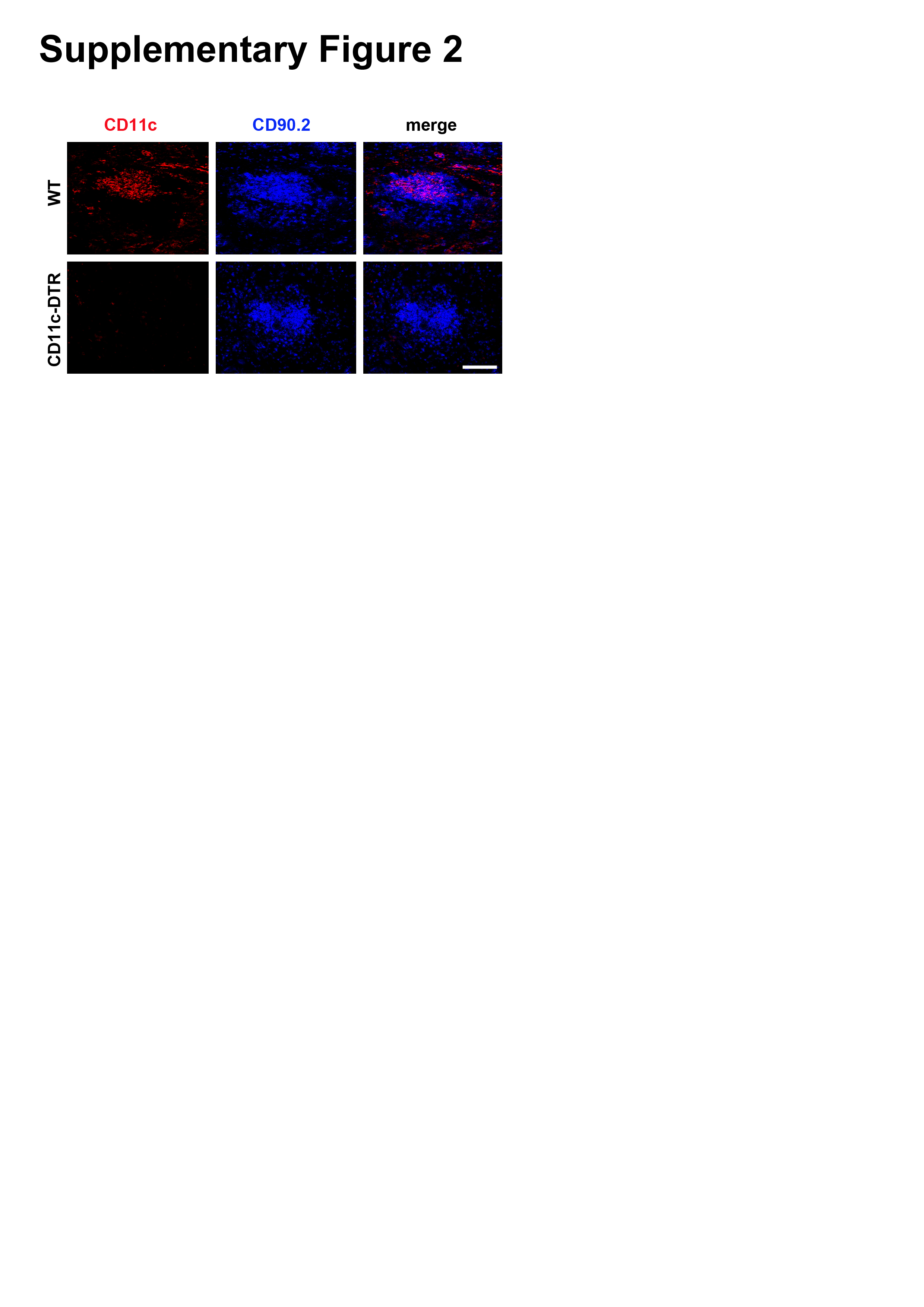

Supplement: Supplementary file 2 [file image_2.jpeg]
